# Imbalance in the Levels of Angiogenic Factors in Patients with Acute and Chronic Central Serous Chorioretinopathy

**DOI:** 10.3390/jcm10051087

**Published:** 2021-03-05

**Authors:** Izabella Karska-Basta, Weronika Pociej-Marciak, Michał Chrząszcz, Agnieszka Kubicka-Trząska, Magdalena Dębicka-Kumela, Maciej Gawęcki, Bożena Romanowska-Dixon, Marek Sanak

**Affiliations:** 1 Department of Ophthalmology, Faculty of Medicine, Clinic of Ophthalmology and Ocular Oncology, Jagiellonian University Medical College, 31-070 Krakow, Poland; weronika.pociej-marciak@uj.edu.pl (W.P.-M.); m.a.chrzaszcz@gmail.com (M.C.); agnieszka.kubicka-trzaska@uj.edu.pl (A.K.-T.); magdalena.debicka-kumela@uj.edu.pl (M.D.-K.); romanowskadixonbozena1@gmail.com (B.R.-D.); 2 Dobry Wzrok Ophthalmological Clinic, 80-402 Gdansk, Poland; maciej@gawecki.com; 3 Molecular Biology and Clinical Genetics Unit, Department of Internal Medicine, Jagiellonian University Medical College Faculty of Medicine, 31-066 Krakow, Poland; marek.sanak@uj.edu.pl

**Keywords:** angiogenesis, angiopoietin-1, central serous chorioretinopathy, proangiogenic factors

## Abstract

Background: The pathogenesis of central serous chorioretinopathy (CSC) remains a subject of intensive research. We aimed to determine correlations between plasma levels of selected angiogenic factors and different forms of CSC. Methods: Eighty patients were enrolled in the study including 30 with a chronic form of CSC, 30 with acute CSC, and 20 controls. Presence of active CSC was determined by fluorescein angiography (FA), indocyanine green angiography (ICGA), and swept-source optical coherence tomography (SS-OCT). Plasma concentrations of angiopoietin-1, endostatin, fibroblast growth factor, placental growth factor (PlGF), platelet-derived growth factor (PDGF-AA), thrombospondin-2, vascular endothelial growth factor (VEGF), VEGF-D, and pigment epithelium–derived factor were measured, and the results were compared between groups. Additionally, mean choroidal thickness (CT) was measured in all patients. Results: Levels of angiopoietin-1 (*p* = 0.008), PlGF (*p* = 0.045), and PDGF-AA (*p* = 0.033) differed significantly between the three groups. Compared with the controls, VEGF (*p* = 0.024), PlGF (*p* = 0.013), and PDGF-AA (*p* = 0.012) were downregulated in the whole CSC group, specifically PDGF-AA (*p* = 0.002) in acute CSC and angiopoietin-1 (*p* = 0.007) in chronic CSC. An inverse correlation between mean CT and VEGF levels was noted in CSC patients (*rho* = −0.27, *p* = 0.044). Conclusions: Downregulated angiopoietin-1, VEGF, PDGF-AA, and PlGF levels may highlight the previously unknown role of the imbalanced levels of proangiogenic and antiangiogenic factors in the pathogenesis of CSC. Moreover, downregulated VEGF levels may suggest that choroidal neovascularization in CSC is associated with arteriogenesis rather than angiogenesis.

## 1. Introduction

Central serous chorioretinopathy (CSC) is a common disease that belongs to the pachychoroid-related disorders, characterized by serous retinal detachment, which is associated with a local leakage from the thicker choroid through impaired retinal pigment epithelium (RPE) [1]. For a long time, a simple classification of CSC into acute and chronic forms has been used; however, it was based solely on the duration of serous neurosensory retinal detachment [2]. Nowadays, as new pathogenic concepts of CSC emerge and modern multimodal imaging becomes available, various new classifications are being developed, but so far, none of them has fully reflected the complexity of this clinical entity [3,4]. Daruich et al. classified CSC as acute, non-resolving, recurrent, chronic, or inactive, which more precisely refers to the course of the disease [5].

As the pathogenesis and pathophysiology of CSC have not been fully explained, the available treatment modalities are suboptimal, especially in long-standing cases [2,6]. Growing evidence indicates that the pathomechanism of CSC is associated with dysfunction of the thickened choroid (an important risk factor), with subsequent impairment of the RPE [1,7]. Some authors have also postulated that downregulation of the cell–cell adhesion molecules in the vascular endothelium could increase the permeability of choroidal vessels, causing fluid leakage under the neurosensory retina [8,9]. In the present study, we attempted to find an association between these phenomena in CSC and the levels of proangiogenic and antiangiogenic factors in human plasma.

Although the role of elevated levels of vascular endothelial growth factor (VEGF) and other cytokines in the pathogenesis of the pachychoroid disease has been speculated on in some papers, they more often referred to other forms of this disease entity than CSC [10,11]. Recent studies suggested that eyes with CSC may be at higher risk of age-related macular degeneration (AMD) or one of its subtypes (polypoidal choroidal vasculopathy or pachychoroid neovasculopathy), or that the development of CSC and AMD/polypoidal choroidal vasculopathy may share a common background [12,13,14]. Data on systemic changes in the expression of proangiogenic and antiangiogenic factors and their role in choroidal vascular homeostasis associated with CSC are scarce. Apart from our previous research, we identified only one report evaluating the plasma level of VEGF in patients with CSC [15,16].

It is well-known that CSC may be complicated by choroidal neovascularization (CNV), a typical feature for neovascular AMD (nAMD). The prevalence of this complication was reported to range from 15.6% to 25% [16,17]. Extensive research of AMD included the levels of proangiogenic factors [18,19,20]. Impaired expression of antiangiogenic factors was shown to play an important role in the development of CNV in the course of nAMD [21]. By analogy, some of these etiological factors typical for nAMD could also be involved in the course of different forms of pachychoroid disease such as CSC. Interestingly, whereas acute exposure to some angiogenic factors such as VEGF results in fast but self-limited hyperpermeability of normal vessels, chronic exposure leads to profound changes in venular function and structure, which results in its chronic hyperpermeability and pathological vessel formation [22]. Angiogenesis and arteriogenesis play a crucial role in tissue development, repair, and regeneration, but also in ocular pathology [23]. Both processes depend on the intricate balance of angiogenic and inflammatory factors [24,25].

To our knowledge, no studies have reported alterations in the levels of circulating angiogenic factors in patients with CSC, which is not an angiogenic condition in itself, but since it is a pachychoroid disease, its phenotype is characterized by attenuation of the choriocapillaris overlying dilated choroidal veins [7]. The purpose of our study was to highlight the previously unknown role of the imbalanced levels of proangiogenic and antiangiogenic factors in CSC. Additionally, our findings may support the search for new therapeutic strategies in CSC and provide new targets for development in the field.

## 2. Materials and Methods

This case-control study included 60 white adult patients (11 women and 49 men) diagnosed with CSC in the Department of Ophthalmology and Ocular Oncology in Kraków, Poland, between November 2017 and June 2018. The control group comprised 20 healthy volunteers from the University Hospital in Kraków, who were matched for sex, age, smoking status, and hypertension. The diagnosis of CSC was based on characteristic findings of indirect ophthalmoscopy, fluorescein angiography (FA), indocyanine green angiography (ICGA, SPECTRALIS, Heidelberg Engineering, Heidelberg, Germany), and SS-OCT (DRI OCT Atlantis, Topcon, Japan). Ocular exclusion criteria were as follows: nAMD, uveitis, diabetic retinopathy, vasculitis, polypoidal choroidal vasculopathy, neovascular glaucoma, anti-VEGF treatment, and other diseases causing macular exudation. Systemic exclusion criteria included any malignancy, acute illness, rheumatoid arthritis, psoriasis, renal or hepatic dysfunction, acute myocardial infarction or stroke within the preceding six months, and corticosteroid treatment. The study was approved by Jagiellonian University Bioethical Committee (Approval No. 122.6120.266.2016), and all patients and controls provided written informed consent to participate in the study.

### 2.1. Clinical Examination

The measurement of best-corrected visual acuity, indirect ophthalmoscopy of the fundus, and SS-OCT were performed in all groups, whereas FA and ICGA were performed only in the CSC group. Central serous chorioretinopathy was classified as acute when symptoms and clinical signs lasted less than six months, whereas chronic CSC was diagnosed when symptoms lasted six months or longer.

The diagnosis of CSC was based on the SS-OCT, FA, and ICGA findings. For SS-OCT, the criteria included current or previous pigment epithelium detachment and/or serous retinal detachment as well as increased CT. On FA, symptoms characteristic for CSC were sought such as focal or multispot dye leakage, dye pooling, or widespread areas of granular hyperfluorescence. Finally, for ICGA, remarkable findings included areas of persistent hyperpermeability during the early and middle phases as well as central hyperfluorescence during the late phase.

Choroidal thickness was assessed using the method previously described by Branchini et al. [26]. Mean CT was considered as the average of the measurements from three points, which were localized beneath the fovea as well as 750 µm temporally and 750 µm nasally from the fovea. The measurements were done by two experienced ophthalmologists (AKT and IKB).

### 2.2. Sample Collection

Blood was drawn from the antecubital vein into BD Vacutainer (BD Life Sciences, Franklin Lakes, NJ, USA) from all participants. Tubes contained (EDTA) as an anticoagulant for plasma preparation. The plasma levels of 10 different angiogenic proteins were measured using the Human Angiogenesis A Premixed Mag Luminex Performance Assay (FCSTM02-10, R&D Systems, Minneapolis, MI, USA). This multiplex immunoassay contains premixed fluorogenic beads with monoclonal antibodies against angiogenin, angiopoietin-1, endostatin, FGF-acidic, FGF-basic, PlGF, PDGF-AA, thrombospondin-2, VEGF, and VEGF-D. The measurements were done according to the manufacturer’s protocol using 1:4 diluted plasma and the xMAP analyzer (Luminex Corporation, Austin, TX, USA). Bead-trapped cytokines were detected by biotin-streptavidin sandwich immunocomplex fluorescence. The results were calculated using 7-point standard curves and proprietary software, Milliplex Analyst Version 5.1 (Merck, Darmstadt, Germany). The plasma levels of angiogenin exceeded the highest concentration of the standard curve calibrator (29,900 pg/mL) in 94% of the samples; therefore, they were not included in further analysis.

### 2.3. Statistical Analysis

Qualitative data were presented as counts and percentages. Quantitative data were shown as means and standard deviations (SD) for normally distributed variables and as medians (Me) and interquartile ranges (IQRs) otherwise. The normality of quantitative variables was tested using the Kolmogorov–Smirnov test. Intergroup comparisons of qualitative variables were made using the Pearson 2 test; this test was used when expected frequencies in more than 80% of cells were higher than five; and the Fisher–Freeman–Halton test was used otherwise. Intergroup comparisons of quantitative variables were made using the 1-way analysis of variance (ANOVA) for normally distributed variables and the Kruskal–Wallis test for variables with nonnormal distribution. When the comparison of the three groups yielded a significant *p* value, a pairwise comparison with Bonferroni correction was used. For FGF-acidic, the ANOVA was used after removing one outlier case present in the acute-CSC group. The CSC (both chronic and acute) and control groups were compared using the t-test or Mann–Whitney test, as appropriate. The results of the comparison were presented graphically with a box plot, where the line inside the box represents a median; the lower and upper sides of the box represent the lower and upper quartiles, respectively; the horizontal lines connected to the box with vertical lines represent cases distant up to 1.5 of the IQR from the respective quartiles; circles represent cases distant from 1.5 to 3 IQRs from the respective quartile; and asterisks represent cases distant by more than 3 IQRs from the respective quartile. The strength of the relationship between quantitative variables was estimated using the Spearman rank correlation coefficient. A *p* value of less than 0.05 was considered significant. The analysis was performed with IBM SPSS Statistics 24 for Windows statistical package.

## 3. Results

In the group with acute CSC (*n* = 30), 83.3% of patients were male compared with 80.0% in the group with chronic CSC (*n* = 30) and 55% in the control group (*n* = 20). The mean (SD) age was 42.7 (9.9) years for patients with acute CSC, 44.5 (6.1) years for those with chronic CSC, and 39.2 (7.4) years for the controls. The demographic and clinical characteristics of the groups are presented in Table 1. Patients with CSC and controls did not differ with regard to age, sex, smoking status, and the prevalence of systemic hypertension. Among the 11 women with CSC, three were after menopause and two used hormonal contraception. Angiotensin-converting enzyme inhibitors were used by four patients with acute CSC, two patients with chronic CSC, and three controls; β-blockers, by one patient with chronic CSC and one control; calcium channel blockers, by four patients with acute CSC and one patient with chronic CSC; diuretics, by six patients with acute CSC, five with chronic CSC, and one control. Finally, sartans were used by one patient with acute CSC and two patients with chronic CSC. Three patients with acute CSC did not use any antihypertensive treatment.

No significant differences were observed in the levels of the studied parameters between patients using at least one antihypertensive drug and those not receiving any antihypertensive medication: PEDF (Me = 94.99, Q1 = 60.99, Q3 = 198.66 and Me = 82.90, Q1 = 47.91, Q3 = 171.66, respectively, *p* = 0.508), FGF-basic (Me = 49.97, Q1 = 48.54, Q3 = 51.37 and Me = 48.54, Q1 = 46.60, Q3 = 51.36, respectively, *p* = 0.234), endostatin (Me = 20,098, Q1 = 16,908, Q3 = 22,640 and Me = 17,684, Q1 = 14,759, Q3 = 19,784, respectively, *p* = 0.05), FGF-acidic (Me = 129.91, Q1 = 125.39, Q3 = 134.43 and Me = 125.39, Q1 = 118.58, Q3 = 129.91, respectively, *p* = 0.067), PDGF-AA Me = 177.81, Q1 = 114.33, Q3 = 256.46 and Me = 191.2, Q1 = 137.72, Q3 = 252.15, respectively, *p* = 0.557), PlGF (Me = 3.24, Q1 = 2.9, Q3 = 4.48 and Me = 3.41, Q1 = 2.9, Q3 = 3.94, respectively, *p* = 0.931), VEGF-D (Me = 79.41, Q1 = 75.32, Q3 = 86.28 and Me = 78.05, Q1 = 72.61, Q3 = 83.52, respectively, *p* = 0.182), thrombospondin-2 (Me = 5534, Q1 = 4455, Q3 = 6725 and Me = 5321, Q1 = 4113, Q3 = 6642, respectively, *p* = 0.553), angiopoetin-1 (Me = 2356, Q1 = 1129, Q3 = 3760 and Me = 2926, Q1 = 1819, Q3 = 3958, respectively, *p* = 0.224), and VEGF (Me = 12.5, Q1 = 5.2, Q3 = 20.5 and Me = 9.8, Q1 = 6.8, Q3 = 16.8, respectively, *p* = 0.968).

The baseline ophthalmological characteristics of patients and controls are presented in Table 2. The groups differed significantly in terms of best-corrected visual acuity and mean CT (Table 2).

At the time of data collection, all patients were treatment naive. Different treatment modalities were applied after plasma samples were collected.

Data on the plasma levels of angiogenic factors in patients with acute and chronic CSC as well as controls are shown in Table 3.

**Figure 1:**
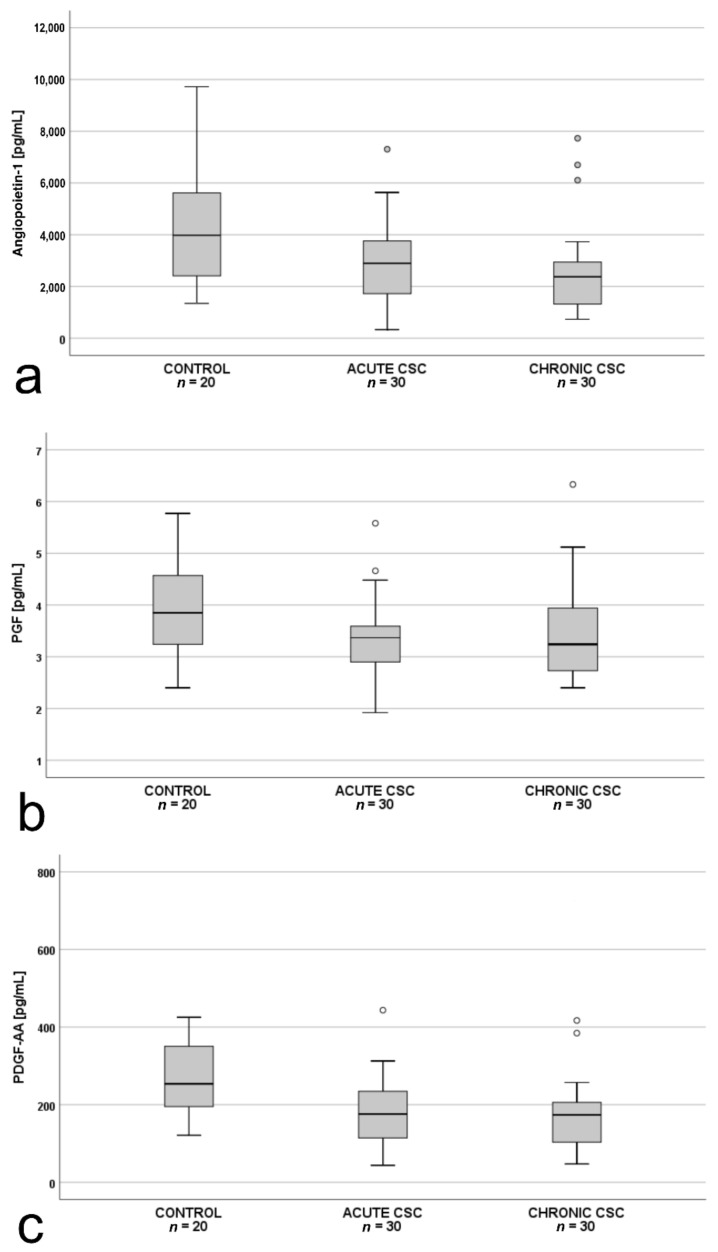
Box-and-whisker plot of: (**a**) plasma angiopoietin-1 (*p* = 0.01; Χ^2^(2) = 9.656); (**b**) placental growth factor (PlGF; *p* = 0.045; F(2.77) = 3.230); and (**c**) plasma pigment epithelium–derived factor (PDGF-AA; *p* = 0.03; F(2.77) = 3.558) levels in patients with acute central serous chorioretinopathy, patients with chronic central serous chorioretinopathy and controls. The line inside the box represents the median; the lower and upper sides of the box represent the lower and upper quartiles, respectively. Whiskers represent cases distant up to 1.5 of interquartile range (IQR) from the respective quartile; circles, cases distant from 1.5 to 3 IQRs. *p* values were estimated using the analysis of variance for normally distributed variables and the Kruskal–Wallis test otherwise.

Intergroup comparisons of the plasma levels of the 10 measured angiogenic factors revealed significant differences between the study groups for angiopoietin-1, PlGF, and PDGF-AA (Figure 1a–c). There were no differences in the levels of the remaining antiangiogenic factors among the three groups (*p* > 0.05).

**Figure 2:**
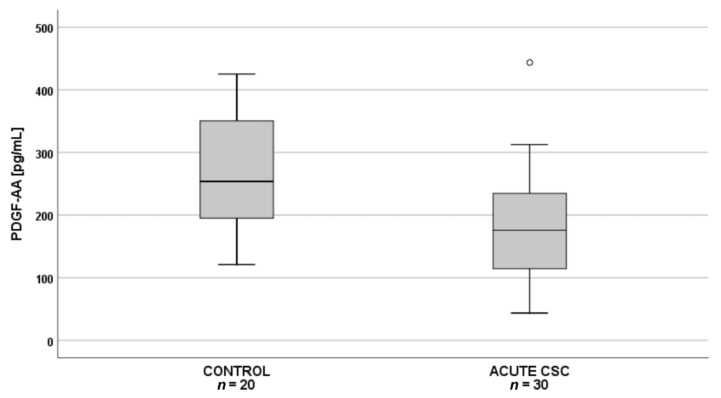
Box-and-whisker plot of plasma pigment epithelium–derived factor (PDGF-AA) levels in patients with acute central serous chorioretinopathy and controls (*p* = 0.002; t(48) = 3.252). The line inside the box represents the median; the lower and upper sides of the box represent the lower and upper quartiles, respectively. Whiskers represent cases distant up to 1.5 of interquartile range (IQR) from the respective quartile; circles, cases distant from 1.5 to 3 IQRs. *p* values were estimated using the analysis of variance post-hoc Bonferroni test.

**Figure 3:**
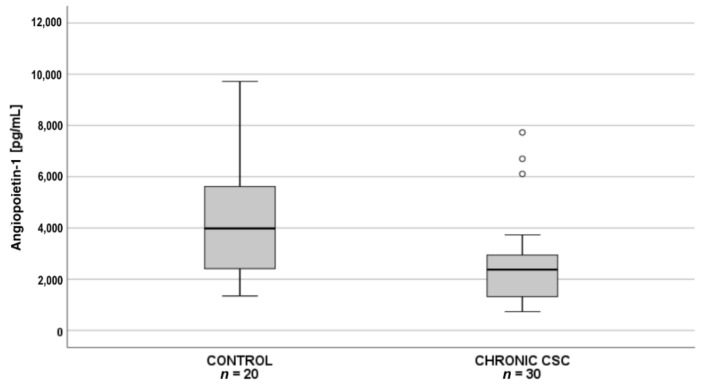
Box-and-whisker plot of plasma angiopoietin-1 levels in patients with chronic central serous chorioretinopathy and controls (*p* = 0.007; Χ^2^(2) = 9.656; post hoc test statistics = 20.3). The line inside the box represents the median; the lower and upper sides of the box represent the lower and upper quartiles, respectively. Whiskers represent cases distant up to 1.5 of interquartile range (IQR) from the respective quartile; circles, cases distant from 1.5 to 3 IQRs. *p* values were estimated using the post-hoc pairwise comparison for the Kruskal–Wallis test with Bonferroni correction.

Subsequent pairwise comparisons showed that in patients with acute CSC (*n* = 30), plasma PDGF-AA levels were significantly lower than in the controls (*n* = 20) (Figure 2), whereas in patients with chronic CSC (*n* = 30), angiopoietin-1 levels were significantly lower than in the controls (*n* = 20) (Figure 3).

**Figure 4:**
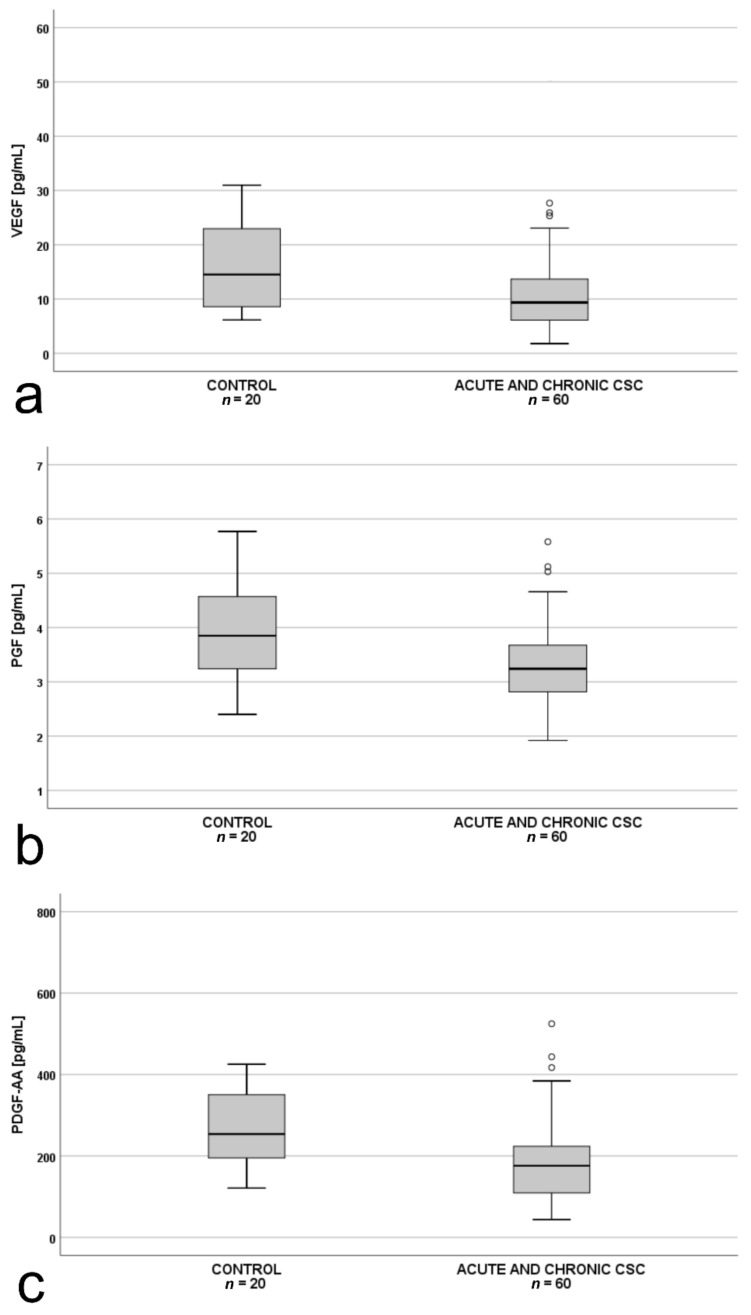
Box-and-whisker plot of: (**a**) plasma vascular endothelial growth factor (VEGF; *p* = 0.024); (**b**) plasma placental growth factor (PlGF; *p* = 0.013; t(48)= −2.551); (**c**) platelet-derived growth factor AA (PDGF-AA; *p* = 0.012; t(48) = −2.577) levels in all patients with central serous chorioretinopathy (acute and chronic) and controls. The line inside the box represents the median; the lower and upper sides of the box represent the lower and upper quartiles, respectively. Whiskers represent cases distant up to 1.5 of interquartile range (IQR) from the respective quartile; circles, cases distant from 1.5 to 3 IQRs. *p* values were estimated using the *t*-test for normally distributed variables and the Mann–Whitney test otherwise.

Additionally, we performed an analysis for the whole CSC group (patients with both chronic and acute form, *n* = 60) vs. controls (*n* = 20). Plasma VEGF, PDGF-AA, and PlGF levels in patients with acute or chronic CSC (*n* = 60) were significantly lower than in the controls (Figure 4a–c).

**Figure 5:**
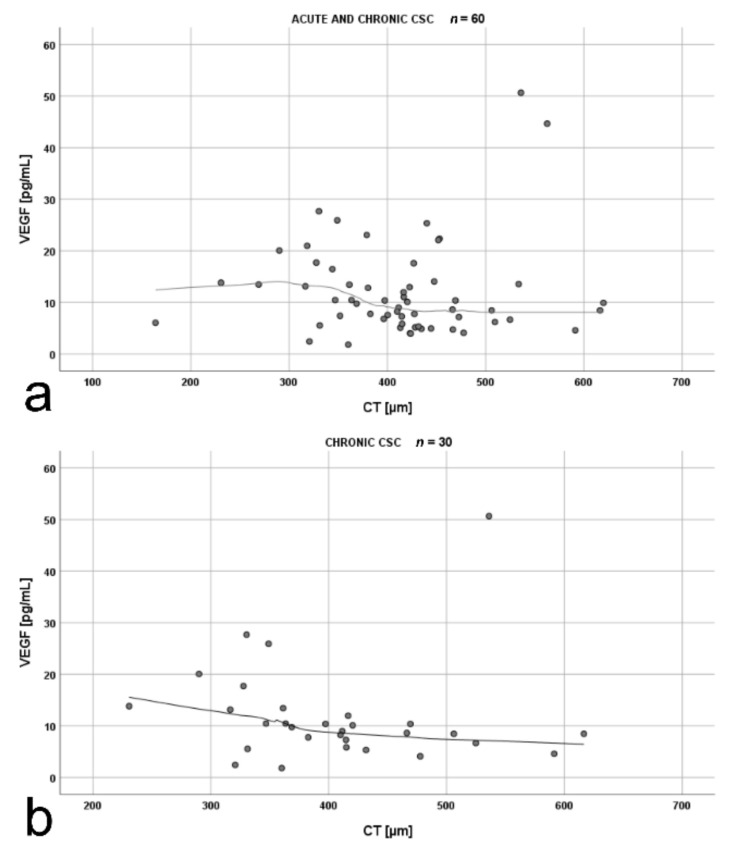
Scatterplots showing the correlations between: (**a**) mean choroidal thickness (CT) and vascular endothelial growth factor (VEGF) levels in patients with acute and chronic central serous chorioretinopathy (*rho =* −0.27, *p* = 0.044); (**b**) mean CT and VEGF levels in patients with chronic central serous chorioretinopathy (*rho =* −0.48, *p* = 0.009). The strength of the relationship between the variables was estimated using the Spearman rank correlation coefficient *rho*. The shape of the relationship was presented using the loess curve.

The analysis of the correlation between mean CT and VEGF for the whole CSC cohort (*n* = 60) revealed an inverse correlation, which was particularly prominent after exclusion of a single outlier in VEGF measurement (>50 pg/mL) (Figure 5a). Further analysis proved that this correlation was true for patients with chronic CSC (Figure 5b), but was not observed in the acute-CSC group (*rho =* 0.07, *p* = 0.721).

## 4. Discussion

Choroidal vasculature plays a crucial role in retinal homeostasis and preservation of good vision and vision-related Quality of Life [27]. Importantly, the key pathophysiologic mechanism in CSC is associated with the presence of abnormally thick choroid, hyperpermeable, and dilated choroidal vessels with or without RPE abnormalities overlying the pachyvessels [7]. Dysregulation in angiogenesis and arteriogenesis has been suggested as an underlying mechanism for the development of some chorioretinal diseases [3,19].

Angiogenesis is a highly controlled process involving the formation of new blood vessels from a preexisting vascular bed and depends on an intricate balance of both proangiogenic and antiangiogenic factors. On the other hand, arteriogenesis refers to anatomic transformation of preexisting arterioles, with an increase in the lumen area and wall thickness, due to a thick muscular layer and development of viscoelastic and vasomotor capacities [25]. The two processes differ in several aspects, with the most important being that angiogenesis depends on hypoxia and arteriogenesis on inflammation [25]. Based on these facts, it may be suspected that there is a relationship between the pathogenesis of pachychoroid diseases, especially CSC, and the levels of angiogenic factors in human blood.

To the best of our knowledge, this is the first study that assessed the plasma levels of angiogenic factors such as angiopoietin-1, endostatin, FGF-acidic, FGF-basic, PlGF, PDGF-AA, thrombospondin-2, VEGF, VEGF-D, and PEDF in patients with CSC in comparison with heathy individuals. We noted differences in the plasma levels of angiopoietin-1, PlGF, and PDGF-AA between patients with acute and chronic CSC as well as the controls. The levels of PDGF-AA were downregulated only in acute CSC, whereas those of angiopoietin-1 only in chronic CSC, compared with the controls.

It has been shown that angiopoietin-1 is essential for vessel stabilization and quiescence in adults [28]. In this context, Lee et al. [29] found that the interendothelial junctional protein reduced recruitment and infiltration of macrophages from the Bruch’s membrane, thus preventing CNV formation and consecutive vascular leakage. Interestingly, a dysregulated interaction between the RPE and infiltrated macrophages results in upregulation of angiogenesis and leads to choroidal abnormalities in chronic CSC [30]. Terao et al. [30] reported that inflammation, accompanied by macrophage infiltration, into the choroid and retina may cause CSC progression from the acute to chronic form. Angiopoietin-1 is also known to support endothelial cell stabilization by activating the Tie-2 receptor and to decrease vascular leakage by increasing the level of the interendothelial cell junction proteins [29]. Previous studies revealed that angiopoietin-1 prevents the VEGF-A–mediated junction disruption [21]; however, a recent report indicated that it may directly stabilize vascular endothelial cadherin and zonula occludens-1 by regulating the RhoA-specific guanine nucleotide exchange factor Syx [31]. Noteworthy, administration of angiopoietin-1 into the vitreous body upregulates the expression of vascular endothelial cadherin and zonula occludens-1, the key factors of endothelial cell-to-cell junctions preserving vascular integrity [28,32]. We hypothesized that the deficiency of angiopoietin-1 demonstrated in our study may result in dissociation of endothelial tight junctions in the choriocapillaris and choroid, leading to chronic vascular dysfunction associated with the prolonged presence of the serous retinal detachment and/or fluid under the RPE in the course of chronic CSC.

Schubert et al. [33] postulated that structural and molecular changes in the choriocapillaris and choroid in CSC can alter the microenvironment of the RPE. This, in turn, can affect RPE barrier capabilities and its transport [33]. The endothelial cells of the choriocapillaris are fenestrated; therefore, they have higher permeability than the nonfenestrated retinal capillaries. This may suggest that even in physiological conditions, the RPE is regularly exposed to plasma filtrate [33]. In our study, the plasma concentrations of PlGF and PDGF-AA were downregulated in patients with CSC compared with the controls. Placental growth factor is a multifunctional cytokine affecting diverse cellular activities [34]. Its pleiotropic effects on junction stabilization, survival, proliferation, and metabolism as well activation effects on vascular cells (i.e., pericytes and smooth muscle cells, endothelial cells) were reported [34]. There is an increasing body of evidence showing that lowering or elevating PlGF expression may lead to various diseases [34]. Moreover, PlGF increases the expression of some angiogenic factors such as VEGF, PDGF-B, and FGF-2 [29], which stimulate angiogenesis through proliferation of endothelial cells and arteriogenesis through smooth muscles [35].

Platelet-derived growth factor stimulates both arteriogenesis and angiogenesis [25]. Tumor growth factor-β produced by different cells is a chemoattractant for monocytes. It also stimulates the expression of PDGF by these cells during arteriogenesis [35], which may be involved in the development of CNV in chronic CSC [36]. The deficiency of PDGF in acute CSC may explain the absence of CNV, but this hypothesis merits further research. Saito et al. [37] confirmed that PDGF-AA was crucial for retina regeneration within the first hours after injury; therefore, its deficiency may play a role in the acute form of CSC.

In our study, the VEGF was significantly downregulated in patients with CSC (both acute and chronic) compared with the controls. Only a few studies assessed angiogenic factors in plasma and aqueous humor (AH) of patients with CSC. Lim et al. [38] did not show any differences in plasma and AH levels of VEGF in CSC compared with the controls. However, the study was limited by a small group of patients, mostly with acute CSC, which precluded a definitive conclusion. Shin et al. [39] reported similar, but very low, AH levels of VEGF in patients with CSC and the controls, but PDGF levels were lower in CSC.

Downregulated plasma VEGF levels in CSC compared with the healthy individuals observed in our study may partially explain an unsatisfactory effect of intravitreal anti-VEGF treatment in patients with CSC [2,40]. Even though CSC is described as a vascular disorder (pachychoroid), the primary exudative component leading to a macular detachment is considered to be nonvasogenic, which means that it does not result directly from the proliferation of choroidal vessels [8,41]. This is an essential difference between CSC and other conditions presenting with serous macular detachment and CNV such as nAMD [8,41,42].

The fact that chronic CSC can be associated with the presence of CNV [16,17] does not contradict the results of our study, which revealed lower plasma VEGF levels in CSC patients compared with the controls. This is in line with the studies by Spaide et al. [36] and Sacconi et al. [43]. They hypothesized that CNV in CSC occurs as a result of proliferation of new vessels during arteriogenesis, which is characterized by dilation of the existing vascular channels and is independent of VEGF (unlike angiogenesis, which is highly VEGF dependent) [44]. On the other hand, VEGF alters the junctional integrity, downregulates the expression of occludin and zonula occludens-1 [45,46,47,48], which results in increased permeability and angiogenesis [15,49]. Downregulated VEGF levels observed in our study may suggest that the mechanism of vascular hyperpermeability in CSC is similar to that of CNV in VEGF independent CSC and may result from altered flow in dilated choroidal vessels (pachyvessels) [7]. The increased flow leads to endothelial cell proliferation, with luminar expansion and release of platelet endothelial cell adhesion molecule-1, monocyte chemoattractant protein-1, intracellular adhesion molecule-1, and vascular cell adhesion molecule-1 [50]. As a result, increased endothelial permeability, as indicated by the leakage of plasma proteins, erythrocytes, and platelets into the vascular wall and the adherence of monocytes to the endothelium, was observed, along with the recruitment of circulating monocytes and resident macrophages [51]. This, in turn, promotes arteriogenesis by the ability of monocytes and macrophages to secrete metalloproteinases, chemokines, and growth factors [25,52].

We hypothesized that the impaired function of RPE cells in CSC, a major source of ocular proangiogenic proteins, may result in decreased intraocular levels of VEGF, but it does not fully explain the downregulation of the other analyzed proangiogenic factors in the eyes, especially in plasma. This issue merits further investigation.

In the current study, we did not find differences in the levels of antiangiogenic proteins, trombospondin-2, and endostatin between patients with CSC and the controls. It is well known that antihypertensive drugs affect angiogenesis [53,54]. However, in our study, we did not observe any differences in the levels of proangiogenic and antiangiogenic factors between patients using at least one antihypertensive drug and those not receiving any medication. This finding requires further research.

In our study, an inverse correlation between mean CT and VEGF was observed in chronic CSC. It was shown that a decrease in VEGF levels in intraocular fluids as a result of intravitreal anti-VEGF therapy leads to a decrease in CT in patients with diabetic retinopathy [55]. Furthermore, some studies reported a decrease in CT in patients with acute CSC after anti-VEGF treatment [56]. These data suggest that certain levels of VEGF are necessary to maintain choroidal stability and function, which is also in line with our results. Nevertheless, this is an interesting phenomenon that definitely deserves a more substantial discussion and more thorough research.

Our study was limited by the fact that it was performed at a single time point and only plasma, and not AH, samples were investigated. Moreover, there were possible confounding factors affecting the levels of angiogenic factors such as concomitant antihypertensive treatment. Finally, during blood sample collection, we did not record the stage of the menstruation cycle in women at reproductive age, while menstruation cycle is known to affect angiogenic markers. Although the lack of AH assessment may be an important limitation, the significant differences in the plasma levels of angiogenic factors are an interesting finding, indicating that CSC might be a systemic disease.

## 5. Conclusions

Downregulated angiopoietin-1, VEGF, PDGF-AA, and PlGF levels observed in our study may highlight the previously unknown role of the imbalanced levels of proangiogenic and antiangiogenic factors, which affect choroidal hyperpermeability and exert profound changes in venular structure and function, thus possibly contributing to the pathogenesis of CSC. Lower plasma levels of VEGF in patients with CSC may support the hypothesis that CNV occurs in these patients as a result of arteriogenesis, which is less VEGF-dependent than angiogenesis. Moreover, our findings may support the search for new therapeutic strategies in CSC. Nevertheless, the underlying mechanisms that contribute to this condition remain largely unknown and require further studies.

## Figures and Tables

**Table 1:** Demographic and clinical characteristics of patients with acute and chronic central serous chorioretinopathy as well as controls.

Variable	Chronic CSC (*n* = 30)	Acute CSC (*n* = 30)	Controls (*n* = 20)	*p* Value
Male sex, *n* (%)	24 (80.0)	25 (83.3)	11 (55.0)	0.056 ^1^
Age, y	44.5 (6.1)	42.7 (9.9)	39.2 (7.4)	0.078 ^2^
Smoking (current, former), *n* (%)	8 (26.7)	7 (23.3)	7 (35.0)	0.658 ^3^
Hypertension, *n* (%)	6 (20.0)	11 (36.7)	4 (20.0)	0.26 ^4^
Hashimoto thyroiditis, *n* (%)	2 (6)	1 (3)	0 (0)	0.781
*Helicobacter pylori* infection, *n* (%)	6 (20)	0 (0)	0 (0)	0.007
Gout, *n* (%)	2 (6)	1 (3)	0 (0)	0.781
Ischemic heart disease, *n* (%)	1 (3)	1 (3)	0 (0)	1.000

^1^ Χ^2^(2) = 5.778; ^2^ F(2.77) = 2.639; ^3^ Χ^2^(2) = 0.836; ^4^ Χ^2^(2) = 2.690. Data were expressed as mean and standard deviation where the analysis of variance was used for comparisons unless specified as number (percentage) using the Pearson χ2 test or the Fisher–Freeman–Halton test as appropriate. A *p* value of less than 0.05 was considered significant. Abbreviations: CSC, central serous chorioretinopathy.

**Table 2:** Ophthalmological characteristics of patients with acute and chronic central serous chorioretinopathy as well as controls.

Variable		Chronic CSC (*n* = 30)	Acute CSC (*n* = 30)	Controls (*n* = 20)	*p* Value
CT, µm		406.1 (88.1)	421.5 (85.3)	317.4 (61.4)	<0.001 ^1^
Affected eye, *n* (%)	Right	10 (33.3)	14 (46.7)	-	0.225 ^2^
	Left	12 (40)	13 (43.3)	-	
	Both	8 (26.7)	3 (10.0)	-	
BCVA(logMAR), *n* (%)	0.3< * ≤0.0	23 (76.7)	20 (66.7)	20 (100.0)	0.017 ^3^
	1.0≤ * ≤0.3	7 (23.3)	10 (33.3)	0	

* BCVA score. ^1^ F(2.76) = 10.770; ^2^ Χ^2^(2) = 2.979; ^3^ Χ^2^(2) = 8.092. Data were expressed as mean and standard deviation where the analysis of variance was used for comparisons unless specified as number (percentage) using the Pearson χ^2^ test or the Fisher–Freeman–Halton test as appropriate. A *p* value of less than 0.05 was considered significant. Abbreviations: BCVA, best corrected visual acuity; CSC, central serous chorioretinopathy; CT, choroidal thickness.

**Table 3:** Plasma levels of angiogenic factors in patients with acute and chronic central serous chorioretinopathy as well as controls.

Angiogenic Factor, pg/mL	Acute CSC (*n* = 30)	Chronic CSC (*n* = 30)	Controls (*n* = 20)	*p* Value
PEDF	94.99 (47.91–200.92)	82.89 (45.73–155.99)	74.12 (54.45–159.35)	0.80 ^1^
FGF-basic	48.97 (2.74)	48.97 (4.71)	50.49 (3.11)	0.28 ^2^
Endostatin	18,009.97 (4466.25)	18,241.17 (4524.54)	16,854.20 (3182.28)	0.49 ^3^
FGF-acid	127.40 (7.92)	125.20 (8.11)	126.08 (8.20)	0.58 ^4^
PDGF-AA	179.04 (87.66)	200.23 (143.25)	265.19 (97.72)	0.03 ^5^
PlGF	3.40 (0.76)	3.44 (0.91)	3.99 (0.96)	0.045 ^6^
VEGF-D	78.62 (5.88)	76.93 (9.66)	77.94 (6.99)	0.70 ^7^
Trombospondin-2	4661.00 (4070.00–6002.00)	5478.00 (5420.00–6933.00)	5898.00 (4750.00–6857.00)	0.07 ^8^
Angiopoietin-1	2894.00 (1722.00–3760.00)	2373.00 (1321.00–2943.00)	3982.00 (2411.00–5617.00)	0.01 ^9^
VEGF	8.80 (6.00-14.40)	9.40 (6.60-13.10)	14.50 (8.60-23.00)	0.07 ^10^

^1^ Χ^2^(2) = 0.447; ^2^ F(2.77) = 1.275; ^3^ F(2.77) = 0.707; ^4^ F(2.76) = 0.553; ^5^ F(2.77) = 3.558; ^6^ F(2.77) = 3.230; ^7^ F(2.77) = 0.359; ^8^ Χ^2^(2) = 5.248; ^9^ Χ^2^(2) = 9.656; ^10^ Χ^2^(2) = 5.091. Data are shown as mean and standard deviation where the analysis of variance was used for comparisons or as median (interquartile range) using the Kruskal–Wallis test for comparison. A *p* value of less than 0.05 was considered significant. Abbreviations: CSC, central serous chorioretinopathy; FGF, fibroblast growth factor; PEDF, pigment epithelium–derived factor; PDGF, platelet-derived growth factor; PlGF, placental growth factor; VEGF, vascular endothelial growth factor.

## Data Availability

Not applicable.
